# Minimal influence of the menstrual cycle or hormonal contraceptives on performance in female rugby league athletes

**DOI:** 10.1002/ejsc.12151

**Published:** 2024-06-15

**Authors:** Ella S. Smith, Jonathon Weakley, Alannah K. A. McKay, Rachel McCormick, Nicolin Tee, Megan A. Kuikman, Rachel Harris, Clare Minahan, Simon Buxton, Jessica Skinner, Kathryn E. Ackerman, Kirsty J. Elliott‐Sale, Trent Stellingwerff, Louise M. Burke

**Affiliations:** ^1^ Mary MacKillop Institute for Health Research Australian Catholic University Melbourne Victoria Australia; ^2^ Sports Performance, Recovery, Injury and New Technologies (SPRINT) Research Centre Australian Catholic University Brisbane Queensland Australia; ^3^ Carnegie Applied Rugby Research (CARR) Centre Carnegie School of Sport Leeds UK; ^4^ Female Performance and Health Initiative Australian Institute of Sport Canberra Australian Capital Territory Australia; ^5^ Perth Orthopaedic and Sports Medicine Research Institute West Perth Western Australia Australia; ^6^ Griffith Sport Science Griffith University Gold Coast Queensland Australia; ^7^ National Rugby League Sydney New South Wales Australia; ^8^ Wu Tsai Female Athlete Program Boston Children's Hospital and Harvard Medical School Boston Massachusetts USA; ^9^ Department of Sport and Exercise Sciences Institute of Sport Manchester Metropolitan University Manchester UK; ^10^ Canadian Sport Institute‐Pacific Victoria British Columbia Canada; ^11^ Exercise Science Physical and Health Education University of Victoria Victoria British Columbia Canada

**Keywords:** estrogen, female, progesterone, sex hormones, strength, women

## Abstract

We examined performance across one menstrual cycle (MC) and 3 weeks of hormonal contraceptives (HC) use to identify whether known fluctuations in estrogen and progesterone/progestin are associated with functional performance changes. National Rugby League Indigenous Women's Academy athletes [*n* = 11 naturally menstruating (NM), *n* = 13 using HC] completed performance tests [countermovement jump (CMJ), squat jump (SJ), isometric mid‐thigh pull, 20 m sprint, power pass and Stroop test] during three phases of a MC or three weeks of HC usage, confirmed through ovulation tests alongside serum estrogen and progesterone concentrations. MC phase or HC use did not influence jump height, peak force, sprint time, distance thrown or Stroop effect. However, there were small variations in kinetic and kinematic CMJ/SJ outputs. NM athletes produced greater mean concentric power in MC phase four than one [+0.41 W·kg^−1^ (+16.8%), *p* = 0.021] during the CMJ, alongside greater impulse at 50 ms at phase one than four [+1.7 N·s (+4.7%), *p* = 0.031] during the SJ, without differences between tests for HC users. Among NM athletes, estradiol negatively correlated with mean velocity and power (*r* = −0.44 to −0.50, *p* < 0.047), progesterone positively correlated with contraction time (*r* = 0.45, *p* = 0.045), and both negatively correlated with the rate of force development and impulse (*r* = −0.45 to −0.64, *p* < 0.043) during the SJ. During the CMJ, estradiol positively correlated to 200 ms impulse (*r* = 0.45, *p* = 0.049) and progesterone to mean power (*r* = 0.51, *p* = 0.021). Evidence of changes in testing performance across a MC, or during active HC use, is insufficient to justify “phase‐based testing”; however, kinetic or kinematic outputs may be altered in NM athletes.

## Highlights

- Evidence of changes in testing performance across a menstrual cycle, or during active hormonal contraceptives use, is insufficient to justify “phase‐based testing” at a group or team‐based level among female rugby league athletes.
- Kinetic or kinematic outputs in jumping movements may be altered in naturally menstruating athletes; however it could not be determined if the observed alterations exceeded between‐day variability.

## 1 INTRODUCTION

Cyclical fluctuations in estrogen and progesterone across the menstrual cycle (MC) potentially influence multiple biological systems associated with athletic performance. Indeed, both sex hormones may influence force development through alterations to muscle contractile properties. Estrogen has been shown to elicit neuroexcitatory effects resulting in increased voluntary activation and reduced inhibition, while progesterone has been shown to exhibit neuroinhibitory effects (Smith et al., 2002). Accordingly, if estrogen and progesterone augment and attenuate force production (Pallavi et al., 2017; Smith et al., 2002) then physical performance may be enhanced when estrogen is elevated and impaired when estrogen is suppressed, with the reverse for progesterone. There is some (albeit predominately low‐quality) evidence for improved force and power outcomes during phases two and four of the MC (when estrogen concentration is high and moderate, respectively), alongside a trivial performance reduction during phase one (when estrogen is low) (McNulty et al., 2020). For women using typical hormonal contraceptives (HC), exogenous estrogen and progestin are supplemented on 21 continuous days and endogenous estrogen and progesterone are therefore suppressed, comparable to the low endogenous hormonal profiles observed during phase one of the MC. Thus, in HC users, there may be marginal performance impairments compared to naturally menstruating (NM) women because of such endogenous estrogen suppression regardless of the daily exogenous estrogen supplementation (Elliott‐Sale et al., 2020).

Cognition is a key aspect of performance in numerous sports, particularly team events that require continuous rapid and accurate decision making. There is a hypothetical role for estrogen and progesterone in cognitive performance based on their entry through the blood–brain barrier and the presence of receptors in multiple brain regions (Brinton et al., 2008; Hara et al., 2015). Indeed, enhanced cognitive performance during MC phase one (low estrogen and progesterone concentrations) has been reported in comparison to other phases involving elevated hormones (Barel et al., 2019; Šimić et al., 2012), which may have relevance to team sports. However this finding is not consistent with other studies reporting no alterations across the MC (Hampson, 1990; Kozaki et al., 2009).

Our understanding of any influence of estrogen or progesterone on physical and/or cognitive performance, through MC phases or with HC use, are inconclusive. This uncertainty partially stems from the broad failure of studies to achieve sufficient methodological classification and control of hormonal profiles (Elliott‐Sale et al., 2020; McNulty et al., 2020). Accurate and purposeful classification of MC phase and HC use is necessary to support causality regarding any influence of estrogen and progesterone on performance. Accordingly, the aim of this study was to examine performance across the MC and between athletes using HC and those with “natural” cycles, employing gold standard protocols regarding the classification and control of participant menstrual status.

## 2 MATERIALS AND METHODS

A comprehensive methodological overview including participant recruitment, study design, and MC tracking is detailed elsewhere (McKay et al., 2023). Only information specific to this study is detailed below.

**FIGURE 1:**
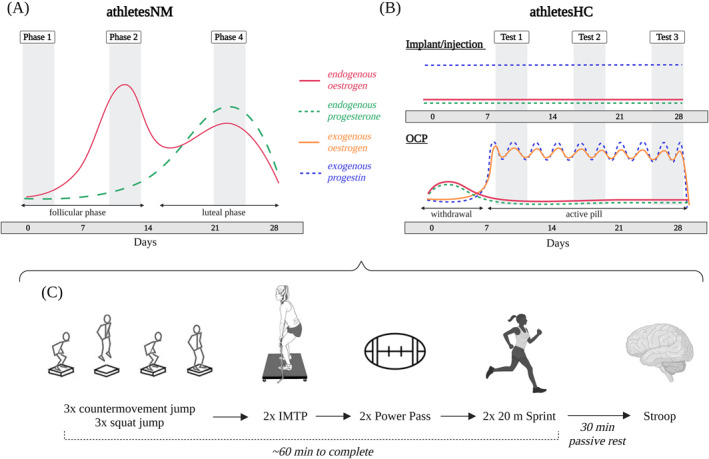
Study overview. Performance testing occurred at either (A) phases one [low estrogen/progesterone concentration (day 1.8 ± 0.4)], two [high estrogen and low progesterone (day 11.4 ± 1.4)] and four [moderate estrogen and progesterone (day 20.8 ± 1.6)] for athletesNM. The days reported refer to the cycle day on which the test was conducted; (B) three equally spaced timepoints for athletesHC utilizing the implant or hormonal injection or, three equally spaced timepoints avoiding the withdrawal bleed for athletesHC using the oral contraceptive pills. It should be noted that the concentration of exogenous progestin following the implant and injection gradually declines with time (Huber, 1998), and hence the exact hormonal profile is dependent on the date of the implant or injection. Time points are displayed according to an idealized 28‐day cycle. (C) Performance testing schedule. Figure created with BioRender.com. HC, hormonal contraceptives; IMTP, isometric mid‐thigh pull; NM, naturally menstruating; OCP, oral contraceptive pills.

Twenty‐four female Tier 3 (national level) (McKay et al., 2022) Australian National Rugby League's Indigenous Women's Academy athletes attended a 5‐week residential training camp at the Australian Institute of Sport. This sample size is reflective of most real‐world rugby squads for which a coach or sports scientist may be asked to consider menstrual phase or status‐based testing at a group level. The group was initially divided into those reporting the use of HC (athletesHC) and those who were considered by their self‐reports as being naturally menstruating (athletesNM) until menstrual status was studied during the project. The actual menstrual status of athletes and their baseline characteristics are summarized in Table 1. This study implemented an observational design within a training camp environment. Following two familiarization sessions, a battery of performance tests was completed on three separate occasions across each participant's individualized menstrual or HC cycle (Figure 1). Participants undertook these tests at the same time of day (±15 min) across a 90 min period, wearing the same shoes, after completing a standardized warm‐up, and adhering to a standardized diet from lunch onwards the day prior to testing (∼18 h). The warm‐up consisted of five minutes cycling on a stationary bike at a perceived “easy” intensity, including 3 × 4 s sprints at 90% of maximal perceived cadence, followed by 10 each of walking lunges, squats, leg swings and calf raises, and concluding with three countermovement jump (CMJ) each at 70% and 90% of perceived maximal effort. For athletesNM, the three phases occurred in a randomized order, determined by the menstrual phase in which they commenced the training camp.

**TABLE 1:** Participant baseline characteristics.

	“Naturally menstruating” (non‐hormonal contraceptive using) athletes (*n* = 11)	Athletes using hormonal contraception (*n* = 13)
Age (yrs)	21 ± 3	22 ± 4
Actual menstrual characteristics	Eumenorrheic (*n* = 1) Naturally menstruating (*n* = 4) Polycystic ovary syndrome (*n* = 1) Oligomenorrheic (*n* = 3) Anovulatory (*n* = 1) Luteal phase deficiency (*n* = 1)	Contraceptive implant (*n* = 8) *[Implanon]* Hormonal injection (*n* = 1) *[Depo Provera]* Combined oral contraceptive pill (*n* = 4) *[Evelyn 150/30 ED:*30 μg *ethinylestradiol,* 150 μg *levonorgestrel, Femme‐Tab 20/100 ED:* 20 μg *ethinylestradiol,* 100 μg *levonorgestrel, Lenest 30 ED:* 30 μg *ethinylestradiol,* 150 μg *levonorgestrel, Yasmin:* 30 μg *ethinylestradiol,* 3 mg *drospirenone]*
Age at menarche (yrs)	13 ± 2	13 ± 2
Body mass (kg)	71.7 ± 8.4	80.1 ± 13.6
Body mass index (kg·m^2^)	27.1 ± 3.4	28.8 ± 4.7

### 2.1 Menstrual status

Menstrual status was tracked in both athletesNM and athletesHC according to best‐practice protocols, (Elliott‐Sale et al., 2021) which included recording the onset of bleeding, conducting 11 weeks of MC or HC tracking, using dual hormone urinary ovulation kits, and assessing retrospective serum 17‐β‐estradiol (the most potent form of estrogen among pre‐menopausal women, henceforth referred to as “estradiol”) and progesterone concentration. Performance testing was completed at MC phases one (day 1.8 ± 0.4), two (day 11.4 ± 1.4), and four (day 20.8 ± 1.6) for athletesNM, and three equally spaced time points for athletesHC (Figure 1). AthletesHC using oral contraceptive pills (OCPs) were tested during pill taking days only and were instructed to take their pill at the same time of day on each testing occasion. As such, AthletesHC were all tested during active HC usage (Test 1, Test 2, and Test 3). Six athletesHC using the contraceptive implant had this inserted between one and 3 years prior to testing and two athletesHC had this inserted the same month as testing commenced. The athleteHC using the hormonal injection had her last injection 3 weeks prior to the first test.

Data presented as mean ± standard deviation (SD). Comprehensive menstrual characteristics are detailed in McKay et al. (2023) (McKay et al., 2023). Menstrual status was defined according to Elliott‐Sale et al. (2021) (Elliott‐Sale et al., 2021)—eumenorrhea: “menstrual cycle length ≥21 days and ≤35 days resulting in 9 or more consecutive periods per year, plus evidence of LH surge, plus correct hormonal profile, plus no HC use 3 months prior to recruitment”, NM “experience menstruation, with MC lengths ≥21 days and ≤35 days, but without confirmed ovulation [ovulation was not confirmed by urinary LH surge or verified by hormone concentrations via blood sample analysis]”, oligomenorrhea: “cycle length >35 days”, anovulatory: “those who experience menstruation but do not ovulate (ovulation cannot be detected by urinary LH surge or confirmed by hormone concentrations via blood sample analysis)”, luteal phase deficiency: “cycles with less than 16 nmol·L^−1^ of progesterone, when a single luteal phase progesterone measurement is taken”.

### 2.2 Blood sampling

Prior to performance testing at each visit, a trained phlebotomist collected an 8.5 mL venous blood sample from an antecubital vein into a serum separator tube while the athlete was in a rested and fasted state. Blood tubes were allowed to clot at room temperature for 30 min and were then centrifuged at 2200 G for 10 min at 4°C. The remaining serum was split into aliquots and stored at −80°C until batch analysis. Estradiol and progesterone were measured via an Access 2 Immunoassay System (Beckman Coulter, Brea, CA, USA) with intra‐assay coefficient of variations (CV) 5% and 11% for estradiol and progesterone, respectively. Total testosterone was analyzed using liquid chromatography‐tandem mass spectrometry (Waters UPLC‐TQX S, Waters Corp.), with a total imprecision CV of 5.8%, and free testosterone was subsequently calculated from total testosterone alongside sex hormone binding globulin and albumin (Vermeulen et al., 1999).

### 2.3 Performance testing protocols

The CMJ, squat jump (SJ), and isometric mid‐thigh pull (IMTP) were conducted on a dual force plate system sampling at 1000 Hz (0.60 × 0.40 m; Model 10 kN 9286B, Kistler Instrument AG, Winterthur, Switzerland). Participants were familiarized at two separate sessions to the CMJ, SJ and IMTP protocols, alongside the Stroop Color and Word Test, during the first 2 days of the training camp. Specific familiarization was not undertaken for the power pass or 20 m sprint as these are regularly performed as part of the National Rugby League testing battery. These tests were selected as they represent different domains of performance (James et al., 2023), were familiar to participants, are commonly used throughout the literature with rugby athletes (Owen et al., 2020), and demonstrate acceptable between‐day reliability and ecological validity (Weakley et al., 2022).

(1) CMJ and SJ

Participants completed three repetitions each of the CMJ and SJ with ∼60 s rest between jumps (Weakley et al., 2022). Participants were instructed to jump as high and powerfully as possible with their hands remaining on hips (both CMJ and SJ). For the SJ, participants jumped from a 90° squat (or as close as possible) without any countermovement. An additional effort was performed if any countermovement was observed. Squat depth was standardized within participants between trials using a plastic pole that participants squatted to reach and touch. The highest jump at each test was taken for analysis; if jump height was equal then peak power was used to determine the “best” effort.

Outcome measures included jump height (calculated through impulse‐momentum), mean and peak concentric force, velocity, and power, alongside impulse and rate of force development (RFD) at 50/100/150/200 ms, as well as contraction time, concentric time, eccentric time, and center of mass displacement. Jump initiation was identified using the criterion method of taking the instant when vertical force was less or greater than a threshold equal to five times the SD of body mass measured during a 1 s stable weighing period (Owen et al., 2014). Jump heights in the CMJ and SJ were also used to calculate the eccentric utilization ratio (EUR) and reactive strength index (RSI), while dynamic strength index (DSI) was calculated from CMJ peak concentric force and IMTP peak force.

(2) Isometric Mid‐Thigh Pull

Following two‐sub maximal warm‐up efforts, participants performed two maximal repetitions of the IMTP separated by 2 min rest. Participants pulled as hard as possible for 3 s on an immovable bar fixed to a customized power rack. Participants were instructed to “push the ground away as hard and as fast as possible”. Verbal encouragement was maintained throughout. A third effort was performed if > 200 N difference was observed between the peak force of the two efforts, there was variability >50 N in the quiet period, there was a countermovement prior to the lift, excessive pre‐tension, or leaning on the bar. The effort with the highest relative peak force was taken for analysis. Initiation of the pull was identified as the moment when force exceeded five SDs of a participant's mass, established through a 1 s stable weighing period. Peak force, time to peak force, RFD, and impulse at 50/100/150/200/250 ms were calculated.

All ground reaction force‐time data for the CMJ, SJ, and IMTP were recorded using ForceDecks software (VALD ForceDecks, 2.0.8587), and then exported for analysis using a customized R script. The kinetic and kinematic outcome variables were selected as they represented different domains of force expression and also provided information that could provide context in relation to changes in temporal performance and movement strategy. Furthermore, ratio data (e.g., DSI) were provided to give context on whether force expression changed relative to difference strength domains (e.g., isometric vs. dynamic strength).

(3) Power Pass

Athletes stood with their feet shoulder‐width apart and pushed a 3 kg med ball from the chest as far as possible into a long‐jump pit. Countermovement in the legs was permitted, but feet were not permitted to leave the ground. The throw distance was measured from the back of the imprint left by the ball in the sand to the nearest cm. The furthest throw at each test was used in analysis.

(4) 20 m Sprint

The 20 m sprint was conducted on an indoor athletics track with four light gates (Fusion SmartSpeed V2) positioned at 0/5/10/20 m, measuring at a height of 57 cm (0 m gate) and 87 cm (5/10/20 m gates). From a split‐stance position, starting 10 cm behind the first light gate as marked‐up on the track (Weakley et al., 2023), participants sprinted at maximal effort for 20 m. The start was initiated when participants broke the plane of the first light gate. An additional light gate, alongside tape to signify a “finishing line”, was placed at ∼23 m. Participants were instructed to run through this line to prevent deceleration prior to 20 m. Each participant completed a warmup sprint, followed by two maximal efforts, with the fastest taken for analysis.

(5) Stroop Color and Word Test

Colored words were displayed on a laptop and participants were asked to indicate the color of the word (not its meaning) by pressing a corresponding key as fast as possible while minimizing errors (Stroop, 1935). Colored labels were placed on keyboard keys to signify the corresponding color. Three types of trials were presented: control (colored rectangles), congruent (words of matched color and meaning), and incongruent (words with mismatched color and meaning). A red “X” flashed onto the screen in the event of an incorrect response. There were 180 trials for each test, taking approximately 3 min to complete. The Stroop test was administered using Inquisit 6 [6.6.1 64bit, (Windows 10), (2020) retrieved from https://www.millisecond.com], in a quiet, private room. The Stroop effect was calculated as the difference between responses (both the proportion correct/accuracy and reaction time) in the incongruent versus congruent trials.

### 2.4 Statistical analyses

Statistical analyses were performed using R Studio (v3.5.2) with statistical significance accepted at an *α* level of *p* ≤ 0.05. Two separate approaches were taken for statistical analyses; participant numbers reported for each outcome measure are displayed in Figure S1. Initially, outcome measures were compared both within individuals (i.e., across menstrual or HC cycle phases) and between individuals (i.e., between athletesNM and athletesHC)—termed “phase‐based analysis”. Linear mixed models were used to analyze each variable, using “menstrual status” and “cycle phase/test day” as fixed effects, alongside “subject identification” and “test order” as random effects. Statistical significance of fixed effects was identified using type II Wald tests with Kenward–Roger degrees of freedom. Where significant fixed effects were established, pairwise comparisons were identified using Tukey *post hoc* adjustments. Non‐normally distributed data were identified using histogram inspection [Stroop outcomes, RFD and impulse during the IMTP, RFD, FT:CT contraction time and concentric time during the CMJ, impulse during the SJ, alongside EUR] and were log transformed prior to statistical analyses. An independent *t*‐test was conducted to compare total training load between groups.

**FIGURE 2:**
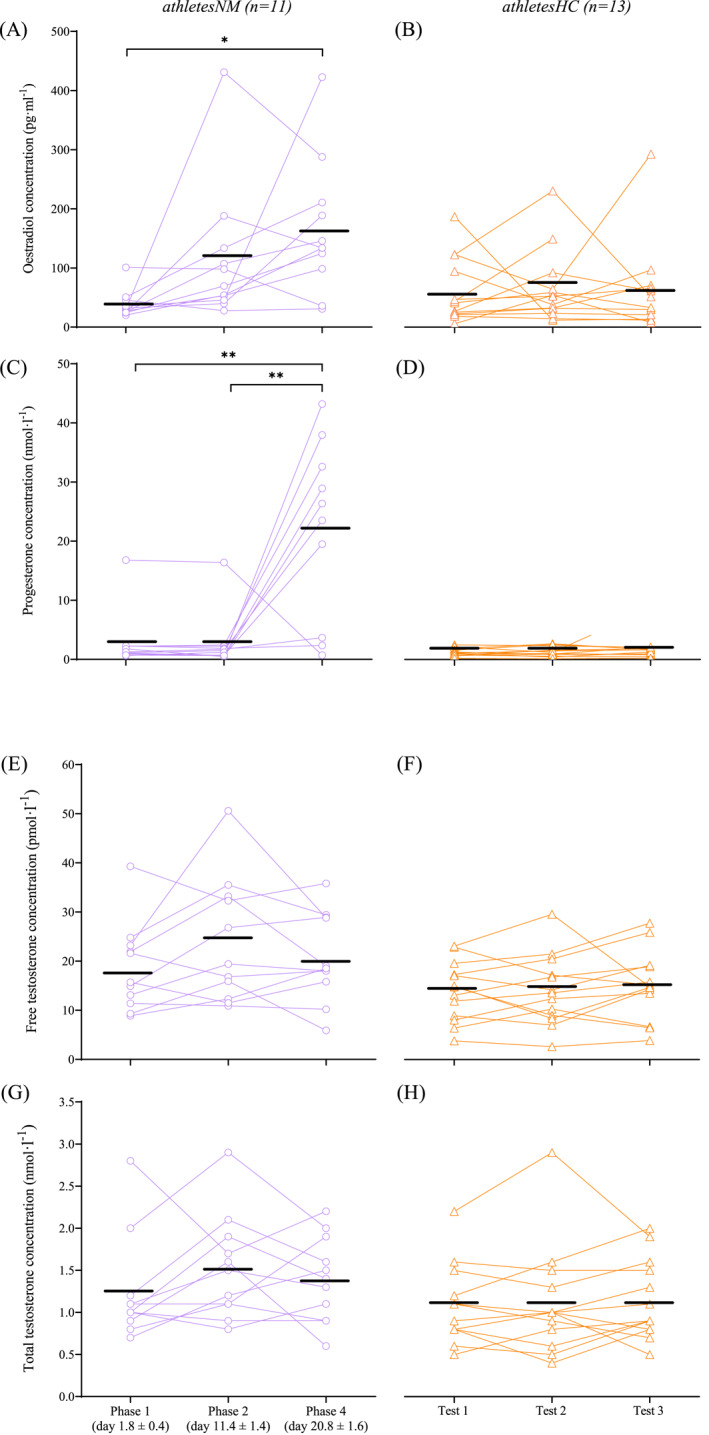
Serum estradiol concentration across the three tests in (A) naturally menstruating athletes and (B) athletes using hormonal contraception (*n* = 1 outlier removed during test three). Serum progesterone concentration across the tests in (C) naturally menstruating athletes (*n* = 1 outlier removed during phase four) and (D) athletes using hormonal contraception. Calculated free testosterone across the tests in (E) naturally menstruating athletes and (F) athletes using hormonal contraception. Total testosterone across the tests in (G) naturally menstruating athletes and (H) athletes using hormonal contraception. Black lines denote mean values. *denotes significance *p* < 0.05, **denotes significance *p* < 0.001.

Following analysis of serum estradiol and progesterone concentrations, it was determined that a “true” phase two was only achieved in one out of 11 athletesNM (McKay et al., 2023) (Figure 2A), and results were therefore compared across phases one and four only. Three athletesNM were also excluded due to hormonal profiles not meeting the criteria for phase four (progesterone >16 nmol·l^−1^, Figure 2C). As such, phase‐based analyses were performed in *n* = 8 athletesNM. Therefore, a repeated measures correlation was also used to assess associations between performance measures and estradiol or progesterone concentration, alongside estradiol to progesterone ratio (E:P) and estradiol to serum free testosterone ratio (E:T)—termed “correlation analysis”. Correlations were conducted among athletesNM exclusively, given that (a) only endogenous hormones were measured and (b) there was potential for variable results outside of hormonal influences due to the largely unknown effects of the exogenous hormonal milieu in athletesHC. This analysis approach did not require discrete MC phases, and thus “phase two” results were included, alongside results from athletes with only two out of three completed tests, resulting in *n* = 11 athletesNM. A single progesterone value from the athlete with PCOS was excluded from correlational analysis because it was >2.5 standard deviations above the mean.

## 3 RESULTS

### 3.1 Hormonal profiles

Among athletesNM, estradiol concentration increased 3‐fold from phase one to “phase two” (*p* = 0.064) and 4.3‐fold from phase one to four (*p* = 0.001, Figure 2A). As a result of the estradiol changes between phases one and two, phase two was only truly captured in one of the 11 athletesNM (McKay et al., 2023). Thus, only phases one and four were analyzed and reported for phase‐based analysis. Progesterone concentration was constant between phases one and “two” (*p* = 0.999), and then increased 8‐fold during phase four (*p* < 0.001, Figure 2C). For athletesHC, endogenous estradiol and progesterone concentrations remained constant across tests (all *p* > 0.05, Figure 2B,D). Both free and total testosterone concentrations were stable across all tests for both groups and did not differ between athletesNM and athletesHC (all *p* > 0.05, Figure 2E,F,G,H). The athlete with the highest estradiol concentration (Figure A) was not the same as that with the highest total testosterone concentration (Figure G).

### 3.2 Performance tests

**FIGURE 3:**
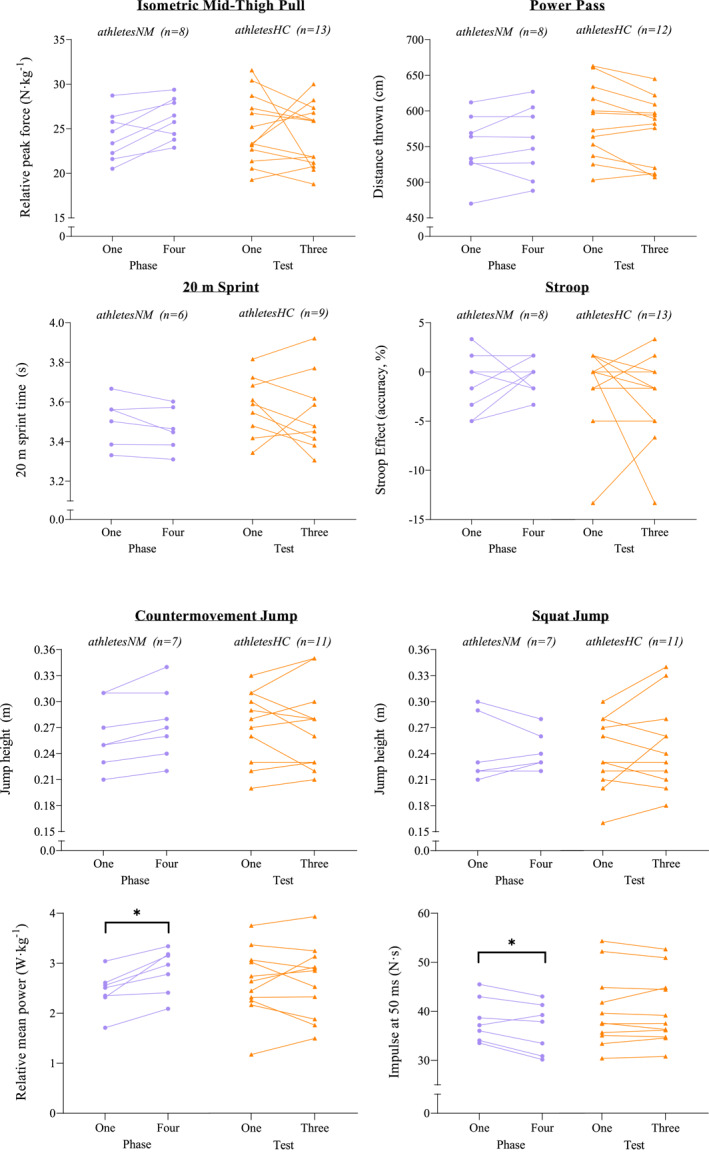
Performance outcomes at each test, alongside relative mean power during the countermovement jump and impulse at 50 ms during the squat jump, between naturally menstruating athletes (athletesNM) and athletes using hormonal contraception (athletesHC). *denotes significance *p* < 0.05.

There was no change in CMJ or SJ height, IMTP peak force, distance thrown in the power pass, fastest sprint time and the Stroop effect between MC phases one and four, or between tests for athletesHC (all p > 0.05, Figure 3), nor any correlation between these outcome measures and estradiol or progesterone concentration among athletesNM. There were also no differences between groups (athletesNM vs. athletesHC) for any performance outcome measure (all p > 0.05). While overall physical performance outcomes were unchanged, there were some small variations in kinetic and kinematic outputs detected during the CMJ and SJ, detailed below. All outcome measures are displayed in the supplementary material (Table S1).

### 3.3 Countermovement jump and squat jump—kinetic and kinematic outcome measures

All outcome measures are displayed in the supplementary material (Table S1). Phase‐based analysis revealed that relative mean concentric power was 16.8% greater in MC phase four than one (*p* = 0.021) among athletesNM during the CMJ (Figure 3), while this remained unchanged between tests in athletesHC (*p* = 1.000). Additionally, athletesNM produced a 4.7% greater impulse at 50 ms in phase one than four (*p* = 0.031) during the SJ (Figure 3), with no change between tests among athletesHC (*p* = 0.999). There were no differences between MC or HC phase for any other outcome measure (*all p* > 0.05), nor there was any difference in calculated metrics *(EUR, RSI, or DSI).*

During the SJ, there were negative correlations between estradiol and RFD at 50 ms (Figure S2A) and 100 ms (*r* = −0.45, *p* = 0.043), as well as between progesterone and RFD at 50 ms (Figure S2B) and 100 ms (*r* = −0.50, *p* = 0.026), but not at 150/200 ms (*p* > 0.05). There were also negative correlations between estradiol and impulse at 50 ms (Figure S2C), 100 ms (*r* = −0.50, *p* = 0.022), and 150 ms (*r* = −0.49, *p* = 0.024), but not at 200 ms (*p* = 0.065), alongside progesterone and impulse at 50 ms (Figure S2D), 100 ms (*r* = −0.56, *p* = 0.011), 150 ms (*r* = −0.53, *p* = 0.016), but not at 200 ms (*p* = 0.067). In addition, there was a negative correlation between estradiol and both mean velocity and relative mean power (Figures S2E,G), and a positive correlation between progesterone and contraction time (Figure S2F). There were negative correlations between E:T and RFD at 50 ms (*r* = −0.49, *p* = 0.023), and impulse at 50 ms (*r* = −0.61, *p* = 0.003), 100 ms (*r* = −0.50, *p* = 0.021), and 150 ms (*r* = −0.46, *p* = 0.036). During the CMJ, we observed positive correlations between estradiol and impulse at 200 ms (Figure S3A), and between progesterone and relative mean power (Figure S3B).

### 3.4 Training load

There were no statistical differences between groups in total training load across the 5 weeks (Table S2).

## 4 DISCUSSION

This study was one of the first to assess a range of performance measures in well‐trained athletes across a MC or during HC use. Our findings indicate that overall physical and cognitive performance outcomes were not statistically different between MC phases one and four in the athleteNM group (*n* = 8), nor across ∼3 weeks within athletesHC (*n* = 13). Furthermore, there were no detectable performance differences between the athleteNM and athleteHC groups. There was also no relationship between overall performance outcomes and estradiol or progesterone concentration among athletesNM. However, despite overall physical performance outcomes being unchanged, some small variations in kinetic and kinematic outputs were detected among athletesNM across the MC in the CMJ and SJ.

While there was no change in jump height, among athetesNM, we observed a 0.41 W·kg^−1^ (16.8%) greater mean concentric power during the CMJ, alongside a 1.7 N·s (4.7%) reduction in impulse at 50 ms during the SJ in phase four compared to phase one (Figure 3), while power and impulse were unchanged between tests for athletesHC. These differences are larger than the intra‐phase CV among athletesNM (9.4% and 2.8% for mean concentric power and impulse at 50 ms, respectively, Table S3), suggesting a true difference in these outcomes between phases. However, these differences were less than the inter‐test CV observed among athletesHC (28.1% and 17.2%, Table S3), and therefore may be attributed to between‐day variability. There were also no differences in performance outcomes between athletesNM and athletesHC. Conversely, a recent meta‐analysis reported trivial strength impairments among women utilizing OCP compared to NM women (Elliott‐Sale et al., 2020). It is possible that differences in the type and mode of hormone delivery of HC used by athletes in the present study [69% using progesterone‐only local HC methods (i.e., implant and injection)] compared to the OCPs examined by Elliott‐Sale et al. (2020) may account for some of this disparity; the effects of different exogenous hormones and absorption routes are largely unknown. If any difference between athletesNM and athletesHC is trivial in magnitude, it may be that the sample size in the present study was too small to detect such differences, or that the differences were too subtle to distinguish, despite testing in an athletic population very familiar with the performance tasks. Indeed, the intra‐test CV for overall performance outcomes among athletesHC ranged from 3.1% to 20.7% (Table S3), and it may be that any small performance differences may have been outweighed by day‐to‐day variability. Taken together, our results currently suggest a lack of justification in the context of altering athlete testing at a group level based solely on menstrual phase or HC use.

In athletesNM, the correlations between estradiol and progesterone concentration and kinetic and kinematic outcomes during the CMJ and SJ are conflicting. Some observations support the hypothesized roles of estradiol and progesterone in augmenting and attenuating neuromuscular function, respectively (Pallavi et al., 2017; Smith et al., 2002). Indeed, during the CMJ, an increase in estradiol concentration was associated with increased impulse at 200 ms (Figure S3A), while during the SJ, elevated progesterone was associated with a decline in RFD and impulse and an increase in contraction time (Figure S2B,D,F). However, in contrast to their hypothesized role, increases in estradiol were simultaneously correlated with a decline in RFD, impulse, mean velocity, and relative mean concentric power during the SJ (Figure S2A,C,E,F), and a progesterone increase was associated with an elevated relative mean power during the CMJ (Figure S3B). The influence of estradiol or progesterone therefore cannot be confirmed. It should also be noted that RFD was highly variable in both intra‐phase and inter‐test (Table S3). A change in bioavailable testosterone between MC phases has also been purported to alter strength/power (Cook et al., 2018), however, free testosterone did not differ across phases among athletesNM. Hence, it appears that the negative relationships between E:T and both early phase RFD and impulse during the SJ are driven by fluctuations in estradiol and not testosterone; and therefore mirror the negative correlations between these outcome measures and estradiol.

The lack of change in overall performance outcomes in athletesNM, or between athletesNM and athletesHC, combined with an inconclusive influence of estradiol and progesterone, suggests that fluctuations in sex hormones may not alter performance outcomes in our population of Tier 3 female athletes. Previous research studies surrounding the effect of MC phase or HC use on performance is highly heterogeneous. Numerous studies support our findings, demonstrating no influence of MC phase on measures of strength, power, or velocity (De Jonge et al., 2001; Lebrun et al., 1995; Romero‐Moraleda et al., 2019). Indeed similar to the present study, both Pessali‐Marques et al. (2024) and Thompson et al. (2021) observed no alteration in CMJ or SJ height between MC phases but did report correlations between both estrogen and progesterone various musculoskeletal parameters (Pessali‐Marques et al., 2024), alongside an enhanced CMJ flight time during phase four compared to two (Thompson et al., 2021). However, there are other reports of improvements in these indices during phases two and three of the MC (Ansdell et al., 2019; Pallavi et al., 2017), as well as a decline in strength‐based outcomes during phase one (Dam et al., 2022; Gordon et al., 2013; McNulty et al., 2020), alongside studies reporting the opposite (Davies et al., 1991; Phillips et al., 1996). Studies examining cognitive performance are similarly inconclusive, with some prior work supporting our lack of relationship between estradiol and progesterone and cognition (Hampson, 1990; Kozaki et al., 2009), while others report alterations across the MC (Barel et al., 2019; Šimić et al., 2012). Therefore, our study of an authentic training squad revealing no detectable differences in overall cognitive or physical performance within or between athletesNM and athletesHC suggests that the logistical difficulties with altering “real‐world” team testing according to MC phase are not justified.

The majority of previous investigations are confounded by a lack of hormonal verification of MC phase or confirmed ovulation (McNulty et al., 2020). This lack of verification hinders the confidence in findings, as the actual phase and hormonal profile at which a measurement has occurred is unknown. Indeed, many studies use the calendar‐based counting approach to classify MC phases, which is demonstrated to be inadequate since it assumes ovulation is exactly mid‐cycle and involves no luteal phase and ovulation assessment (Elliott‐Sale et al., 2021). Moreover, due to intra‐individual MC variability, a particular cycle day is not guaranteed to be the same phase in different cycles in the same individual (Elliott‐Sale et al., 2021). Prior studies also examined different combinations of “phases” (e.g., two vs. four, follicular vs. luteal) consequently hindering the ability to compare findings across studies.

The methodological quality of MC control and phase verification may influence study findings. The meta‐analysis by McNulty et al. (2020) reported that the majority of papers (12 out of 13) demonstrating differences in strength between MC phases were of low quality, while those studies identified as moderate‐to‐high‐quality trended toward no differences between MC phase (nine out of 10). Training status may also impact any influence of MC phase on performance. Differences on performance indices examined may be too subtle to detect in an athletic population that is already highly trained in the performance indices examined. Hormonal influence may not exceed typical day‐to‐day performance variability, or differences are masked by high training volumes. Indeed, prior studies examining participants ≥ Tier 2 (McKay et al., 2022) in combination with some MC phase verification (retrospective serum estradiol and progesterone and/or confirmed ovulation) have typically trended toward null findings pertaining to alterations in strength/power/speed across MC phases (Julian et al., 2017; Lebrun et al., 1995; Romero‐Moraleda et al., 2019; Vaiksaar et al., 2011). In addition, prior studies have typically examined performance tasks that lack applicability to a high performance sporting environment, such as single‐limb exercises (McNulty et al., 2020), whereas our study utilized common performance measures, including those utilized in the National Rugby League testing battery. It may be that the higher performance variability in the dynamic sport‐specific tests examined in the present study versus controlled or lab‐based tasks also outweighed small differences in performance across the MC. The higher athletic caliber of our participants, the sport‐specific ecological validity, combined with gold standard classification and control of menstrual status, may therefore help to explain the lack of performance differences between phases.

Other factors that may influence performance should also be considered. For example, pre‐menstrual symptoms commonly associated with the end of phase four or beginning of phase one may alter performance, irrespective of any hormonal influences (e.g., cramps, bloating, tiredness, gastrointestinal issues, and poor sleep). These negative symptoms are reportedly experienced by ∼60%–93% of female athletes (Findlay et al., 2020; Martin et al., 2018), with ∼50%–67% believing that such symptoms impair performance (Bruinvels et al., 2017; Findlay et al., 2020). However, we observed a low frequency of symptoms throughout the duration of the training camp, as assessed through daily online questionnaires reporting symptom presence (McKay et al., 2023). Thus, MC symptoms appear unlikely to have influenced performance. However, symptom severity was not recorded and so presents an area for future study.

Study findings should be considered in light of potential limitations. Phase two could only be confirmed in one out of the 11 athletesNM. While highlighting the complexities of research among women, this also meant that a correlational approach was taken to facilitate the inclusion of “phase two” data, which is unable to determine causality. Measurements of serum estradiol and progesterone were collected at a single timepoint on the day of testing, meaning it was not possible to determine if the hormonal concentration was increasing or decreasing. Moreover, diurnal variation in endogenous estradiol and progesterone concentrations were also not considered. While we acknowledge our study, with its small participant number, may be underpowered to detect marginal differences in our chosen performance tests, this is one of the first studies using sport‐specific performance tasks among well‐trained (Tier 3) athletes with a gold‐standard approach to MC classification and control, thus improving the robustness and ecological‐validity of our findings to the athlete‐specific literature. Given the well‐trained nature of the population in a training camp environment, it was not possible to control training load in the days preceding testing, which may have masked the ability to detect any small performance alterations. Additionally, testing occurred across a single MC among athletesNM, so we could not determine if any observed effects prevailed during another MC. Since such limitations are also present in the real world, when coaches or performance scientists are asked to consider regimens involving menstrual phase or status‐based testing at a group level for a squad, particularly in a national team camp environment, we feel that our study outcomes are still able to inform a decision regarding phase‐based testing. The participant cohort also presented a heterogeneous mixture of hormonal profiles, with the athletesHC group using a variety of HC types and menstrual irregularities detected among six athletesNM, which may also have obstructed the detection of any minor performance alterations. However, the divide between athletesHC (54%) and athletesNM (46%) is similar to the reported prevalence rates among athletes (Martin et al., 2018), and therefore reflective of heterogeneity within a real‐world training squad for which a coach might be asked to consider “menstrual phase or status” testing programs. Our findings suggests that such an approach is not justified at the group level. However, in the applied setting it may be beneficial to undertake long‐term MC tracking on an individual athlete basis to identify any performance alterations with menstrual status, although such repeated and longitudinal measures were beyond the scope of the present study.

## 5 CONCLUSIONS AND FUTURE RESEARCH

Our findings demonstrate no detectable influence of MC phase or HC use on overall physical and cognitive performance outcomes among rugby league athletes. Some kinetic or kinematic outputs during jumping movements may be altered; however, it could not be determined if the observed alterations exceeded between‐day variability. Further research is required to determine causality and fully understand the effects of estradiol and progesterone on performance, alongside underpinning mechanisms. In the meantime, our study represents a real‐world training squad for which a coach might be asked to consider “menstrual phase‐ or status‐based” testing programs and fails to provide evidence that such an approach is justified at a team‐based level.

## CONFLICT OF INTEREST STATEMENT

Authors declare no conflict of interest.

## ETHICAL STATEMENT

Ethical approval was granted by the Australian Catholic University Human Ethics Research Committee (2021‐285H) in accordance with the Declaration of Helsinki. The study was approved by the Australian Catholic University Human Ethics Research Committee (2021‐285H) in accordance with the Declaration of Helsinki.

## Supporting information

Supporting Information S1

## Data Availability

The data that support the findings of this study are available from the corresponding author upon reasonable request.
