# Supplementary material for: Minimal influence of the menstrual cycle or hormonal contraceptives on performance in female rugby league athletes
Source: Eur J Sport Sci. 2024 Jun 15;24(8):1067–78. doi: 10.1002/ejsc.12151 (PMC11295101; doi:10.1002/ejsc.12151)
Supplement: Supplementary file 1 — Supporting Information S1 [file EJSC-24-1067-s001.docx]

**Supplementary material**

**Figure S1.**

**
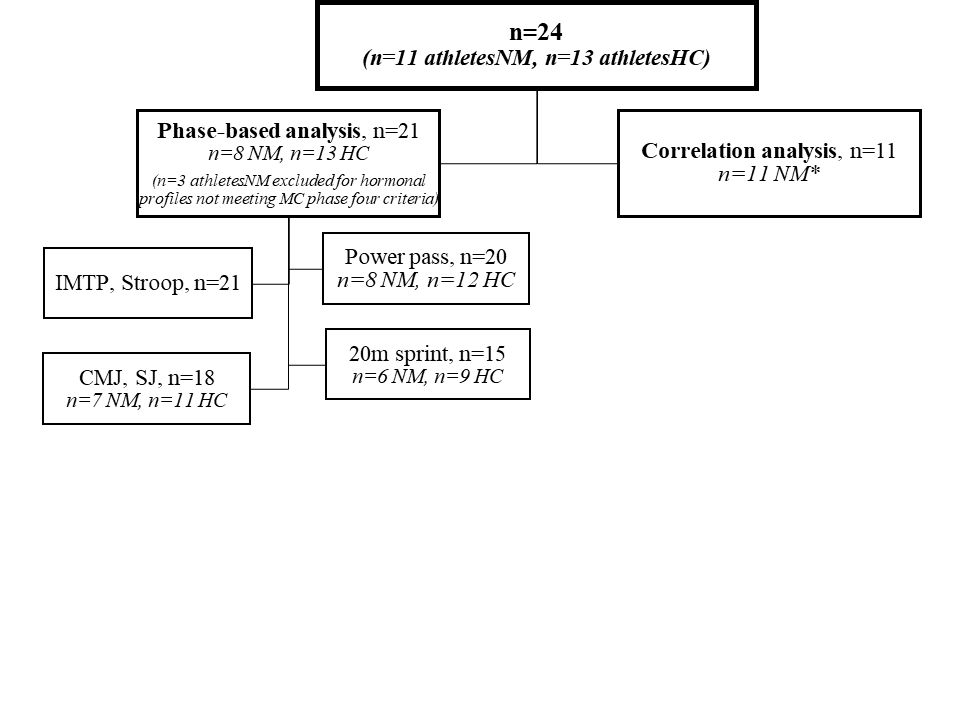
**

**Figure S1.** Flow chart illustrating the participant number reported for each outcome measure for both phase-based and correlational analysis approaches. Athletes excluded for each variable were due to sickness/injury. *a single progesterone value was excluded from correlational analysis because it was >2.5 standard deviations above the mean. NM; naturally menstruating, HC; hormonal contraception.

**Figure S2.**

A)

B)

C)

D)

E)

G)

F)

**Figure S2.** Repeated measures correlations during the squat jump between rate of force development at 50 ms and (A) estradiol and (B) progesterone, between impulse at 50 ms and (C) estradiol and (D) progesterone, between (E) mean velocity and estradiol, (F) contraction time and progesterone, (G) relative mean power and estradiol. Correlations among naturally menstruating athletes (n=11). The different color lines and symbols represent different participants.

**Figure S3.**

A)

B)

**Figure S3.** Repeated measures correlations during the countermovement jump between (A) impulse at 200 ms and estradiol, and (B) relative mean power and progesterone. Correlations among naturally menstruating athletes (n=11). The different color lines and symbols represent different participants.

**Table S1.** Outcome measures assessed across all performance tests among naturally menstruating athletes and athletes using hormonal contraception.

| Performance Test | Outcome measure | Naturally menstruating athletes (n=11) | | Athletes using hormonal contraception (n=13) | |
| --- | --- | --- | --- | --- | --- |
|  |  | Phase one | Phase four | Test one | Test three |
| Countermovement Jump | Jump height (m) | 0.26 ± 0.04 | 0.27 ± 0.04 | 0.27 ± 0.05 | 0.27 ± 0.04 |
|  | Velocity at take-off (m·s^-1^) | 2.27 ± 0.16 | 2.31 ± 0.17 | 2.32 ± 0.18 | 2.31 ± 0.21 |
|  | Relative peak force (N·kg^-1^) | 21.9 ± 1.36 | 22.8 ± 1.49 | 21.8 ± 1.69 | 22.3 ± 2.27 |
|  | Relative mean force (N·kg^-1^) | 12.6 ± 0.3 | 12.8 ± 0.3 | 12.6 ± 0.6 | 12.7 ± 0.6 |
|  | Relative peak power (W·kg^-1^) | 42.2 ± 4.7 | 43.3 ± 5.2 | 43.5 ± 5.1 | 43.6 ± 5.4 |
|  | Relative mean power (W·kg^-1^) | **2.4 ± 0.4** | **2.9 ± 0.5*** | 2.6 ± 0.7 | 2.6 ± 0.7 |
|  | Peak velocity (m·s^-1^) | 2.39 ± 0.15 | 2.43 ± 0.16 | 2.45 ± 0.16 | 2.44 ± 0.19 |
|  | Mean velocity (m·s^-1^) | 1.27 ± 0.04 | 1.30 ± 0.07 | 1.31 ± 0.09 | 1.31 ± 0.11 |
|  | RFD at 50 ms (N·s^-1^) | -1517 ± 1517 | -1515 ± 2219 | -1150 ± 1241 | -615 ± 2235 |
|  | RFD at 100 ms (N·s^-1^) | -1674 ± 1479 | -1577 ± 1812 | -1444 ± 1153 | -1113 ± 1949 |
|  | RFD at 150 ms (N·s^-1^) | -1152 ± 1278 | -1269 ± 1176 | -1057 ± 961 | -1043 ± 1460 |
|  | RFD at 200 ms (N·s^-1^) | -1162 ± 1472 | -1998 ± 2681 | -1359 ± 1846 | -1690 ± 2399 |
|  | RFD minimum-maximum (N·s^-1^) | 3974 ± 1513 | 4373 ± 1866 | 3933 ±1588 | 4212 ± 1643 |
|  | Impulse at 50 ms (N·s) | 71.5 ± 10.3 | 73.8 ± 11.1 | 77.8 ± 11.8 | 78.8 ± 12.9 |
|  | Impulse at 100 ms (N·s) | 139.7 ± 19.0 | 144.1 ± 21.2 | 153.2 ± 23.9 | 155.7 ± 22.7 |
|  | Impulse at 150 ms (N·s) | 205.4 ± 28.5 | 212.1 ± 33.4 | 225.7 ± 34.1 | 229.5 ± 30.3 |
|  | Impulse at 200 ms (N·s) | 270.8 ± 33.7 | 276.1 ± 38.0 | 297.2 ± 42.8 | 299.3 ± 37.1 |
|  | Total impulse (N·s) | 356.2 ± 54.7 | 348.2 ± 46.4 | 390.7 ± 74.5 | 384.1 ± 75.5 |
|  | Flight time: contraction time (s) | 0.60 ± 0.08 | 0.65 ± 0.06 | 0.53 ± 0.19 | 0.61 ± 0.12 |
|  | Contraction time (s) | 0.83 ± 0.10 | 0.76 ± 0.07 | 0.84 ± 0.15 | 0.82 ± 0.17 |
|  | Concentric time (s) | 0.54 ± 0.08 | 0.49 ± 0.05 | 0.56 ± 0.12 | 0.54 ± 0.14 |
|  | Eccentric time (s) | 0.28 ± 0.04 | 0.27 ±0.04 | 0.29 ± 0.05 | 0.28 ± 0.04 |
|  | Centre of mass displacement (m) | -0.30 ± 0.05 | -0.29 ± 0.05 | -0.31 ± 0.06 | -0.30 ± 0.07 |
|  |  |  |  |  |  |
| Squat Jump | Jump height (m) | 0.24 ± 0.04 | 0.24 ± 0.02 | 0.24 ± 0.04 | 0.25 ± 0.05 |
|  | Velocity at take-off (m·s^-1^) | 2.19 ± 0.15 | 2.18 ± 0.09 | 2.17 ± 0.19 | 2.21 ± 0.23 |
|  | Relative peak force (N·kg^-1^) | 19.9 ± 1.4 | 19.9 ± 1.5 | 20.4 ± 2.3 | 20.2 ± 1.9 |
|  | Relative mean force (N·kg^-1^) | 15.0 ± 0.6 | 14.7 ± 0.6 | 14.6 ± 0.9 | 14.7 ± 1.0 |
|  | Relative peak power (W·kg^-1^) | 39.8 ± 3.9 | 39.8 ± 3.3 | 40.4 ± 5.6 | 41.1 ± 6.2 |
|  | Relative mean power (W·kg^-1^) | 14.2 ± 1.5 | 13.2 ± 0.9 | 12.7 ± 2.3 | 13.3 ± 2.7 |
|  | Peak velocity (m·s^-1^) | 2.32 ± 0.14 | 2.31 ± 0.09 | 2.31 ± 0.17 | 2.36 ± 0.21 |
|  | Mean velocity (m·s^-1^) | 0.95 ± 0.09 | 0.89 ± 0.05 | 0.86 ± 0.14 | 0.89 ± 0.15 |
|  | RFD at 50 ms N·s^-1^) | 3258 ± 1287 | 2063 ± 1471 | 2237 ± 1811 | 2176 ± 1389 |
|  | RFD at 100 ms (N·s^-1^) | 3479 ± 901 | 2576 ± 1352 | 2635 ± 1836 | 2855 ± 1681 |
|  | RFD at 150 ms (N·s^-1^) | 2912 ± 920 | 2440 ± 634 | 2270 ± 1233 | 2552 ± 1257 |
|  | RFD at 200 ms (N·s^-1^) | 2213 ± 953 | 2071 ± 666 | 1896 ± 826 | 2084 ± 915 |
|  | RFD minimum-maximum (N·s^-1^) | 2213 ± 935 | 1984 ± 634 | 2169 ± 681 | 2129 ± 629 |
|  | Impulse at 50 ms (N·s) | **38.3 ± 4.5** | **36.6 ± 5.1*** | 40.0 ± 7.5 | 40.0 ± 7.0 |
|  | Impulse at 100 ms (N·s) | 86.8 ± 11.7 | 80.8 ± 14.3 | 88.1 ± 20.4 | 88.1 ± 17.7 |
|  | Impulse at 150 ms (N·s) | 141.9 ± 20.0 | 131.3 ± 23.6 | 141.7 ± 34.7 | 143.8 ± 31.6 |
|  | Impulse at 200 ms (N·s) | 199.7 ± 31.1 | 186.2 ± 32.4 | 198.2 ± 48.6 | 201.7 ± 44.7 |
|  | Total impulse (N·s) | 443.5 ± 50.9 | 457.9 ± 52.7 | 508.5 ± 71.9 | 510.8 ± 72.9 |
|  | Flight time: contraction time (s) | 1.11 ± 0.11 | 0.92 ± 0.38 | 1.00 ± 0.18 | 1.01 ± 0.19 |
|  | Contraction time (s) | 0.42 ± 0.04 | 0.44 ± 0.05 | 0.46 ± 0.06 | 0.46 ± 0.08 |
|  |  |  |  |  |  |
| Isometric Mid-Thigh Pull | Relative peak force (N·kg^-1^) | 24.2 ± 2.7 | 26.1 ± 2.3 | 24.9 ± 3.8 | 24.2 ± 3.6 |
|  | Time to peak force (s) | 2.7 ± 1.5 | 3.6 ± 1.1 | 3.0 ± 1.2 | 2.4 ± 0.8 |
|  | RFD at 50 ms (N·s^-1^) | 2909 ± 2127 | 3179 ± 2643 | 1349 ± 2255 | 2549 ± 2995 |
|  | RFD at 100 ms (N·s^-1^) | 3013 ± 2261 | 3336 ± 2357 | 1307 ± 2239 | 2563 ± 2842 |
|  | RFD at 150 ms (N·s^-1^) | 3206 ± 2458 | 3471 ± 2268 | 1196 ± 1185 | 2615 ± 2448 |
|  | RFD at 200 ms (N·s^-1^) | 3032 ± 2297 | 3481 ± 1941 | 1129 ± 1600 | 2527 ± 1970 |
|  | RFD at 250 ms (N·s^-1^) | 2491 ± 2125 | 3205 ± 1521 | 1226 ± 1357 | 2260 ± 1655 |
|  | Impulse at 50 ms (N·s) | 57.9 ± 17.2 | 49.3 ± 11.3 | 50.6 ± 9.8 | 59.9 ± 18.7 |
|  | Impulse at 100 ms (N·s) | 123.1 ± 34.0 | 107.8 ± 27.8 | 104.5 ± 20.2 | 126.0 ± 38.5 |
|  | Impulse at 150 ms (N·s) | 197.0 ± 50.3 | 174.8 ± 47.8 | 161.1 ± 33.5 | 198.9 ± 59.3 |
|  | Impulse at 200 ms (N·s) | 262.4 ± 66.3 | 233.4 ± 63.8 | 214.8 ± 43.5 | 264.6 ± 77.8 |
|  | Impulse at 250 ms (N·s) | 363.9 ± 81.8 | 334.4 ± 96.2 | 283.0 ± 63.2 | 361.2 ± 99.4 |
|  |  |  |  |  |  |
| Calculated metrics | Eccentric Utilization Ratio | 1.13 ± 0.06 | 1.16 ± 0.08 | 1.16 ± 0.13 | 1.12 ± 0.08 |
|  | Reactive Strength Index | 3.57 ± 1.65 | 4.27 ± 2.02 | 3.61 ± 2.20 | 2.85 ± 1.60 |
|  | Dynamic Strength Index | 0.58 ± 0.10 | 0.58 ± 0.05 | 0.59 ± 0.06 | 0.56 ± 0.07 |
|  |  |  |  |  |  |
| Power Pass | Distance thrown (m) | 5.49 ± 0.45 | 5.56 ± 0.50 | 5.86 ± 0.52 | 5.72 ± 0.48 |
|  |  |  |  |  |  |
| 20 m Sprint | Time to 5m (s) | 1.18 ± 0.04 | 1.17 ± 0.03 | 1.25 ± 0.07 | 1.23 ± 0.07 |
|  | Time to 10m (s) | 2.02 ± 0.07 | 2.00 ± 0.05 | 2.11 ± 0.13 | 2.08 ± 0.10 |
|  | Time to 20m (s) | 3.50 ± 0.12 | 3.46 ± 0.11 | 3.66 ± 0.27 | 3.58 ± 0.20 |
|  |  |  |  |  |  |
| Stroop Colour and Word Test | Stroop effect accuracy (%) | -1.3 ± 3.1 | -0.4 ± 1.7 | -1.3 ± 4.0 | -3.4 ± 4.3 |
|  | Stroop effect reaction time (ms) | 93.2 ± 24.3 | 107 ± 63.5 | 151 ± 93.0 | 141 ± 93.0 |

*Results presented as mean ± standard deviation. *significantly different from menstrual cycle phase one (p<0.05). RFD; rate of force development.*

**Table S2.** Mean total training load across the five-week training camp.

|  | Naturally menstruating athletes (n=11) | Athletes using hormonal contraception (n=13) | *p value* |
| --- | --- | --- | --- |
| Training duration (hours) | 32.3 ± 4.4 | 32.1 ± 5.1 | *0.944* |
| RPE x duration (AU) | 10,782 ± 2,144 | 10,572 ± 3,210 | *0.856* |
| Distance covered in field sessions (km) | 30.6 ± 13.4 | 34.6 ± 7.3 | *0.387* |
| Distance covered in field sessions per minute played (m·min^-1^) | 346 ± 128 | 385 ± 80 | *0.390* |
| Gym volume load (AU) | 38,519 ± 9,388 | 40,122 ± 7,436 | *0.645* |

*Results presented as mean ± standard deviation.*

**Table S3.** Mean intra-phase coefficient of variation for naturally menstruating athletes and inter-test coefficient of variation for athletes using HC for performance outcome measures and kinematic outputs.

| Performance Test | Outcome measure | Naturally menstruating athletes (n=11) | Athletes using hormonal contraception (n=13) |
| --- | --- | --- | --- |
|  |  | Intra-phase CV (%) | Inter-test CV (%) |
| Countermovement Jump | Jump height (m) | 4.0 | 16.6 |
|  | Velocity at take-off (m·s^-1^) | 1.9 | 8.5 |
|  | Relative peak force (N·kg^-1^) | 3.2 | 9.6 |
|  | Relative mean force (N·kg^-1^) | 1.3 | 4.6 |
|  | Relative peak power (W·kg^-1^) | 2.2 | 13.0 |
|  | Relative mean power (W·kg^-1^) | 9.4 | 28.1 |
|  | Peak velocity (m·s^-1^) | 1.4 | 7.2 |
|  | Mean velocity (m·s^-1^) | 2.5 | 8.1 |
|  | RFD at 50 ms (N·s^-1^) | 338.2 | 213.9 |
|  | RFD at 100 ms (N·s^-1^) | 4.0 | 131.1 |
|  | RFD at 150 ms (N·s^-1^) | 54.6 | 119.7 |
|  | RFD at 200 ms (N·s^-1^) | 34.9 | 126.6 |
|  | RFD minimum-maximum (N·s^-1^) | 17.5 | 37.3 |
|  | Impulse at 50 ms (N·s) | 3.6 | 15.3 |
|  | Impulse at 100 ms (N·s) | 3.0 | 14.1 |
|  | Impulse at 150 ms (N·s) | 2.6 | 13.0 |
|  | Impulse at 200 ms (N·s) | 2.2 | 12.4 |
|  | Total impulse (N·s) | 2.4 | 19.4 |
|  | Flight time: contraction time (s) | 6.1 | 36.3 |
|  | Contraction time (s) | 5.1 | 17.8 |
|  | Concentric time (s) | 7.0 | 21.1 |
|  | Eccentric time (s) | 4.3 | 16.1 |
|  | Centre of mass displacement (m) | 5.6 | 19.2 |
|  |  |  |  |
| Squat Jump | Jump height (m) | 5.3 | 20.7 |
|  | Velocity at take-off (m·s^-1^) | 2.7 | 10.4 |
|  | Relative peak force (N·kg^-1^) | 1.7 | 10.5 |
|  | Relative mean force (N·kg^-1^) | 2.4 | 6.4 |
|  | Relative peak power (W·kg^-1^) | 2.9 | 15.1 |
|  | Relative mean power (W·kg^-1^) | 8.4 | 19.6 |
|  | Peak velocity (m·s^-1^) | 2.3 | 8.6 |
|  | Mean velocity (m·s^-1^) | 6.9 | 15.8 |
|  | RFD at 50 ms N·s^-1^) | 43.5 | 71.8 |
|  | RFD at 100 ms (N·s^-1^) | 35.4 | 62.1 |
|  | RFD at 150 ms (N·s^-1^) | 24.5 | 49.2 |
|  | RFD at 200 ms (N·s^-1^) | 17.7 | 40.7 |
|  | RFD minimum-maximum (N·s^-1^) | 7.7 | 29.8 |
|  | Impulse at 50 ms (N·s) | 2.8 | 17.2 |
|  | Impulse at 100 ms (N·s) | 4.8 | 19.7 |
|  | Impulse at 150 ms (N·s) | 5.7 | 20.8 |
|  | Impulse at 200 ms (N·s) | 5.6 | 20.8 |
|  | Total impulse (N·s) | 3.5 | 16.3 |
|  | Flight time: contraction time (s) | 10.8 | 25.6 |
|  | Contraction time (s) | 5.7 | 14.3 |
|  |  |  |  |
| Isometric Mid-Thigh Pull | Relative peak force (N·kg^-1^) | 4.7 | 15.2 |
|  | Time to peak force (s) | 39.6 | 38.1 |
|  | RFD at 50 ms (N·s^-1^) | 66.8 | 123.4 |
|  | RFD at 100 ms (N·s^-1^) | 52.1 | 111.1 |
|  | RFD at 150 ms (N·s^-1^) | 51.2 | 99.4 |
|  | RFD at 200 ms (N·s^-1^) | 51.3 | 86.7 |
|  | RFD at 250 ms (N·s^-1^) | 55.3 | 78.6 |
|  | Impulse at 50 ms (N·s) | 10.3 | 24.8 |
|  | Impulse at 100 ms (N·s) | 11.6 | 24.8 |
|  | Impulse at 150 ms (N·s) | 12.4 | 25.1 |
|  | Impulse at 200 ms (N·s) | 12.2 | 24.7 |
|  | Impulse at 250 ms (N·s) | 13.0 | 25.2 |
|  |  |  |  |
| Power Pass | Distance thrown (m) | 3.0 | 8.1 |
|  |  |  |  |
| 20 m Sprint | Time to 5m (s) | 1.3 | 6.3 |
|  | Time to 10m (s) | 0.8 | 6.0 |
|  | Time to 20m (s) | 0.7 | 6.7 |
|  |  |  |  |
| Stroop Color and Word Test | Stroop effect accuracy (%) | N/A | 3.1 |
|  | Stroop effect reaction time (ms) | N/A | 75.8 |

*Results presented as mean ± standard deviation. Intra-phase CV for the Stroop Test is not applicable for athletesNM as only one test was completed per phase (i.e., no repeats). CV; coefficient of variation*
